# Fluorouracil exacerbates alpha-crystallin B chain—mediated cell migration in triple-negative breast cancer cell lines

**DOI:** 10.1038/s41598-023-31186-7

**Published:** 2023-03-10

**Authors:** Lili Yang, Yuya Haga, Akihide Nishimura, Yuki Tsujii, Suzuno Tanahashi, Hirofumi Tsujino, Kazuma Higashisaka, Yasuo Tsutsumi

**Affiliations:** 1 Graduate School of Pharmaceutical Sciences, Osaka University, 1-6 Yamadaoka, Suita, Osaka, 565-0871 Japan; 2 The Museum of Osaka University, 1-13 Machikaneyama, Toyonaka, Osaka, 560-0043 Japan; 3 Institute for Advanced Co-Creation Studies, Osaka University, 1-6 Yamadaoka, Suita, Osaka, 565-0871 Japan; 4 Global Center for Medical Engineering and Informatics, Osaka University, 2-2 Yamadaoka, Suita, Osaka, 565-0871 Japan

**Keywords:** Breast cancer, Cell migration

## Abstract

Among triple-negative breast cancer (TNBC) subtypes, the basal-like 2 (BL2) subtype shows the lowest survival rate and the highest risk of metastasis after treatment with chemotherapy. Research has shown that αB-crystallin (*CRYAB*) is more highly expressed in the basal-like subtypes than in the other subtypes and is associated with brain metastasis in TNBC patients. We therefore hypothesized that αB-crystallin is associated with increased cell motility in the BL2 subtype after treatment with chemotherapy. Here, we evaluated the effect of fluorouracil (5-FU), a typical chemotherapy for the treatment of TNBC, on cell motility by utilizing a cell line with high αB-crystallin expression (HCC1806). A wound healing assay revealed that 5-FU significantly increased cell motility in HCC1806 cells, but not in MDA-MB-231 cells, which have low αB-crystallin expression. Also, cell motility was not increased by 5-FU treatment in HCC1806 cells harboring stealth siRNA targeting *CRYAB*. In addition, the cell motility of MDA-MB-231 cells overexpressing αB-crystallin was significantly higher than that of MDA-MB-231 cells harboring a control vector. Thus, 5-FU increased cell motility in cell lines with high, but not low, αB-crystallin expression. These results suggest that 5-FU–induced cell migration is mediated by αB-crystallin in the BL2 subtype of TNBC.

## Introduction

Triple-negative breast cancer (TNBC) accounts for 15–20% of all breast cancer cases^1^. Compared with other types of breast cancer, TNBC shows a shorter survival time, higher rate of mortality within five years after diagnosis, and higher rate of distant metastasis^2^. Because TNBC lacks hormone receptors (estrogen receptor and progesterone receptor) and human epidermal growth factor receptor 2 (HER2)^3^, therapies targeting these receptors are not an option, and cytotoxic chemotherapy agents such as fluorouracil (5-FU)^4^, cisplatin (CDDP)^5^, and paclitaxel (PXT)^6^ are used as first-line treatments. However, sensitivity to chemotherapy in TNBC patients varies among individuals, resulting in variations in the efficacy of neoadjuvant chemotherapy and survival rate^7^. For instance, neoadjuvant chemotherapy increases the rate of pathologic complete response in about 20% of TNBC patients, but other patients show a significantly worse 3-year survival^4,8^.

Based on tumor gene expression profile, TNBC patients are divided into six subtypes: basal-like 1, basal-like 2 (BL2), mesenchymal, mesenchymal stem-like (MSL), immunomodulatory, and luminal androgen receptor^9^. When treated with chemotherapy, the BL2 subtype shows the lowest survival rate and the MSL subtype shows the highest^10^. The BL2 subtype also shows the shortest disease-free time and has the highest risk of metastasis after chemotherapy^11^. Thus, how BL2-subtype cells react to chemotherapeutic agents is an important topic of research.

Despite the efficacy of neoadjuvant chemotherapy, PXT increases the risk of breast cancer metastasis by activating the tumor microenvironment of metastasis and increasing its density^12^. However, it is currently unclear whether chemotherapeutic agents affect cell motility. Research has shown that αB-crystallin (*CRYAB*) is required for cell migration and invasion in HER2-positive breast cancer cells^13^ and is more highly expressed in the basal-like TNBC subtypes than in the other subtypes^14,15^. Furthermore, clinical analyses of TNBC patients have revealed that αB-crystallin expression is associated with poor survival after brain metastasis^16^. In addition, we have shown that HCC1806 cells (BL2 subtype) have greater capacity for migration and express more *CRYAB* than do MDA-MB-436 cells (MSL subtype), suggesting that αB-crystallin may contribute to the migration of HCC1806 cells^17^. Based on these findings, we hypothesized that αB-crystallin is involved in the low response to chemotherapy of the BL2 subtype by increasing cell motility. Here, we examined the role of αB-crystallin in the response of TNBC cells to treatment with the typical chemotherapy agent 5-FU.

## Materials and methods

### Cell lines and cell cultures

Three breast adenocarcinoma cell lines HCC1806, MDA-MB-231, and MDA-MB-436 were purchased from American Type Culture Collection (ATCC, Manassas, VA, USA). HCC1806 cells were cultured in RPMI-1640 (Wako, Osaka, Japan); MDA-MB-231 and MDA-MB-436 cells were cultured in DMEM (Wako). Both culture media were supplemented with 10% (v/v) inactivated fetal bovine serum (FBS; Biosera, Nuaille, France) and 1% (v/v) penicillin–streptomycin-amphotericin B suspension (Fujifilm Wako Pure Chemical, Osaka, Japan). Cells were maintained at 37 °C under 5% CO_2_ in air.

### Cell viability

HCC1806, MDA-MB-231, and MDA-MB-436 cells were seeded at 2 × 10^4^ cells per well in 96-well flat plates (Thermo Fisher Scientific, Waltham, MA, USA) and incubated overnight at 37 °C under 5% CO_2_ in air. Then, the cells were treated with 5-FU (Wako) (dose range, 0.05–50 μM) for 24 h. The 5-FU was dissolved in dimethyl sulfoxide and stored at − 80 °C until use. The drug doses refer to final concentrations and were achieved by diluting the stock solution with cell culture medium. Cell viability was evaluated by using a WST-8 assay kit (Nacalai Tesque, Kyoto, Japan).

### Wound healing assay

Each cell line was seeded at 1.5 × 10^5^ cells/well in 24-well flat plates (Thermo Fisher Scientific) and incubated overnight at 37 °C under 5% CO_2_ in air. The cell was treated with 5-FU for 24 h, and then a scratch was made in the cell layer by using a sterile micropipette tip. The cell layer was then washed with phosphate-buffered saline (PBS) and incubated for a further 24 h in culture medium without FBS. Images of the scratch area were acquired at the beginning and end of the 24-h incubation period. The images were recorded using a light microscope (IX71N-22, Olympus, Tokyo, Japan) or an all-in-one microscope BZ-X800 (Keyence Engineering Corporation, Osaka, Japan), and were analyzed by using ImageJ software (version 1.6.0_24, National Institutes of Health, Bethesda, MD, USA). The reduction rate of the scratch area was calculated as follows:

$$\begin{aligned} {\text{Relative healing area }} & = \, \left( {{\text{wound area after scratch }} - {\text{ wound area after incubation}}} \right) \, / \, \\ & \;\;\;\left( {\text{wound area after scratch}} \right) \, \times \, 100\% \\ \end{aligned}$$

### RNA interference (short interfering RNA and short hairpin RNA)

HCC1806 cells were transfected with 10 nM Stealth siRNA targeting *CRYAB* (5ʹ-CCCUCUCACCAUUACUUCAtt-3ʹ and 3ʹ-UGAAGUAAUGGUGAGAGGGtc-5ʹ) or Stealth siRNA negative control with medium GC content (Invitrogen, Carlsbad, CA, USA). The transfection was executed by adding Lipofectamine RNAiMAX Transfection Reagent (Invitrogen) to the cell culture medium for 48 h. To generate stable shRNA cells, pLKO.1-puro vector cloned with a targeted shRNA sequence (TRCN0000010823, sh*CRYAB*#1: CCGGCCGTGAAGAGAAGCCTGCTGTCTCGAGACAGCAGGCTTCTCTTCACGGTTTTT or TRCN0000003842, sh*CRYAB*#2: CCGGTCCCTGAGTCCCTTCTACCTTCTCGAGAAGGTAGAAGGGACTCAGGGATTTTT) was provided by the Center for Medical Research and Education, Graduate School of Medicine, Osaka University. To generate virus for transduction, HEK293T cells (ATCC) were transfected with the cloned vector and the packaging plasmids PsPAX.2 (Addgene, Watertown, MA, USA) and pMD2.G (Addgene), with Fugene (Promega, Madison, WI, USA). HCC1806 cells were infected with the virus supernatant and selected with 2 μg/mL puromycin (Sigma-Aldrich) for further analysis. The stable cell lines harboring sh*CRYAB* or negative control shRNA were named sh*CRYAB*#1, sh*CRYAB*#2, and shCtrl, respectively.

### Immunoblotting analysis

Proteins were extracted from cells by using M-PER Mammalian Protein Extraction Reagent with Halt Protease and Phosphatase Inhibitor Single-Use Cocktail (100 ×), and protein concentrations were evaluated by using a Pierce BCA Protein Assay Kit (all from Thermo Fisher Scientific). A 10-µg portion of each protein sample was mixed with 0.167-volume of 6 × western blot loading buffer that consisted of 60% (w/v) bromophenol blue, 12% (w/v) sodium dodecyl sulfate (SDS), and 60% (w/v) glycerol dissolved in sterile distilled water containing 0.375 M Tris–HCl and 600 mM dithiothreitol (all from Wako). Then, the samples were boiled for 5 min prior to separation by SDS polyacrylamide gel electrophoresis. Precision Plus Protein Kaleidoscope molecular weight markers (Bio-Rad Laboratories, Hercules, CA, USA) were used as standards. The proteins were then electro-transferred onto a polyvinylidene difluoride membrane (Millipore, Bedford, MA, USA) and blocked for 1 h at room temperature in 5% (w/v) skim milk powder (Wako) diluted in Tris-buffered saline containing 0.01% (v/v) Triton X-100 (5% skim milk-TBST) for αB-crystallin, or in 4% (w/v) Block Ace (DS Pharma Biomedical, Osaka, Japan) diluted in phosphate-buffered saline containing 0.01% (v/v) Triton X-100 (4% Block Ace-PBST) for other proteins. The membranes were incubated with primary antibody overnight at 4 °C and then treated with secondary antibody for 1 h. The following antibodies were used: anti-αB-crystallin monoclonal antibody (D6S9E, 1:1000) and anti-rabbit IgG-horseradish peroxidase-conjugated secondary antibody (1:2000) purchased from Cell Signaling Technology (Danvers, MA, USA); anti-α-tubulin monoclonal antibody (AC-74, 1:1000) and anti-mouse IgG (whole molecule)–peroxidase secondary antibody (NA.46, 1:50,000) purchased from Sigma-Aldrich. The protein bands on the membranes were detected with ImmunoStar LD (Wako) and visualized with an ImageQuant LAS 4000 mini biomolecular imager (GE Healthcare Japan, Tokyo, Japan). Band intensity was quantified by film densitometry using ImageJ software. The intensity of each protein band was normalized to that of β-actin.

### Transwell migration assay

Each cell line was seeded at 4 × 10^5^ cells/well in 6-well plates and incubated overnight at 37 °C under 5% CO_2_ in air. Then, 5-FU was diluted with cell culture medium and added to the cell medium to a final concentration of 0.05, 0.5, or 5 μM for 24 h. The cells were then washed and resuspended in serum-free medium and added at 1 × 10^5^ cells/well to Transwell inserts containing 8-μm pore polycarbonate filters (Corning, Lowell, MA, USA) for 24 h at 37 °C under 5% CO_2_ in air. Cells remaining in the upper chamber were removed with a moist cotton swab. Cells that had migrated to the lower side of the chamber were fixed, stained with 0.1% crystal violet (Sigma-Aldrich, St Louis, MO, USA), and examined under a BZ-X800 all-in-one microscope (Keyence Engineering Corporation). The number of cells that migrated to the lower side of the membrane were counted by using the ImageJ software.

### Stable cell line with CRYAB overexpression

The pCMV3-Flag-CRYAB plasmid encoding human *CRYAB* and pCMV3 negative control vectors were purchased from Sino Biological (Beijing, China). The pCMV3-Flag-CRYAB and pCMV3 negative control vectors were separately transfected into MDA-MB-231 cells at 70%–80% confluency with Fugene (Promega) for 24 h in DMEM containing 10% FBS. Then, the cells were selected by culturing in D-MEM containing 10% FBS with 50 μg/mL of hygromycin (Nacalai Tesque) for 2 weeks. Stable cell lines with *CRYAB* overexpression (MDA-MB-231 OEαB cells) or with the pCMV3 negative control vectors (MDA-MB-231 OENC cells) were used for further analysis.

### Statistical analysis

Statistical analyses were conducted using Graph Pad Prism Mac (version 9.0; GraphPad Software, La Jolla, CA; www.graphpad.com). Results are expressed as mean ± SD. Differences were compared by using two-way analysis of variance (ANOVA) followed by post-hoc Tukey’s HSD test. *P* < 0.05 was considered statistically significant.

## Results

### 5-FU induces cell migration in HCC1806 cells

**Figure 1:**
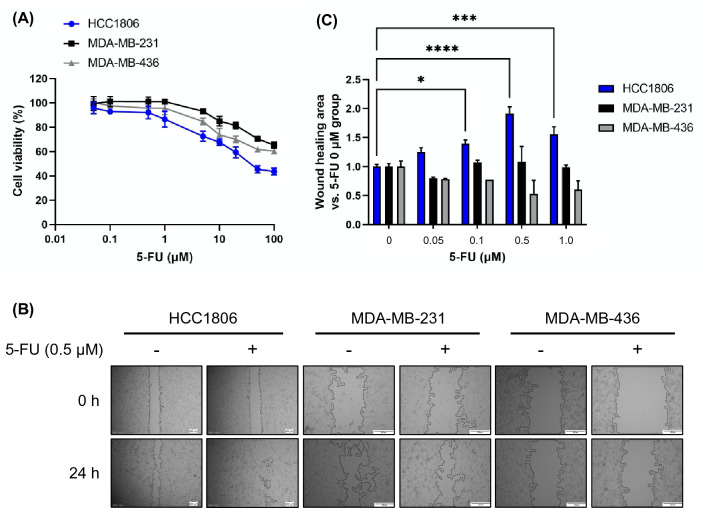
Fluorouracil (5-FU) increased cell mobility in HCC1806 cells. (**A**) Cells were treated with 0.1–100 μM 5-FU for 24 h and cell viability was determined by WST-8 assay. Data are represented as mean ± SD; n = 5. (**B**) Cells were seeded in 24-well plates. A scratch was made in the cell monolayer and then the monolayer was treated with 0.5 μM 5-FU for 24 h. Images of the wound area at the beginning and end of the treatment period were recorded. Scale bars = 200 μm. (**C**) Cells were seeded in 24-well plates. A scratch was then made in the cell monolayer and the monolayer was treated with 0.05–1 μM 5-FU for 24 h. ImageJ software was used to calculate the relative healing area. Data are presented as mean ± SD; n = 3. *P < 0.05; ***P < 0.001; ****P < 0.0001. Results are representative of two independent experiments.

First, αB-crystallin expression was analyzed in several cell line of TNBC subtypes. The result shows that the expression level of αB-crystallin in basal-like subtypes was greater than that in other subtypes of breast cancer and in fibrosarcoma cell line HT1080 (Fig. S1). Meanwhile, HCC1806 cell line has the highest expression level of αB-crystallin among the cells. Therefore, HCC1806 cells (high αB-crystallin expression) were used to represent the BL2 subtype and as controls; MDA-MB-231 cells and MDA-MB-436 cells (low αB-crystallin expression) were used to represent the MSL subtype. The effect of 5-FU on cell viability by means of a WST-8 assay. In all three cell lines, cell viability remained above 80% at 5-FU concentrations of 1 μM and below (Fig. 1A); therefore, a concentration range of 0.05 to 1 μM was used in a wound healing assay to examine the effect of 5-FU on cell migration. Cell migration was significantly increased by 5-FU treatment at concentrations in the range of 0.1–1 μM in HCC1806 cells compared with that in the untreated group (Fig. 1B,C). In contrast, no change in cell motility was observed in MDA-MB-231 and MDA-MB-436 cells.

The cell viability and migration experiments were then repeated with two other common chemotherapies, CDDP and PXT (Fig. S2). CDDP inhibits DNA synthesis via a mechanism similar to that of 5-FU^18,19^, whereas PXT works by suppressing microtubule dynamic stability^20^. Cell viability remained above 80% at CDDP concentrations of 1 μM and below (Fig. S2A) and at PXT concentrations of 2 mM and below (Fig. S2B). Cell migration was significantly increased in HCC1806 cells treated with 0.5 or 1 μM CDDP compared with the untreated control (Figure S2C and S2D). In contrast, cell migration was significantly decreased in HCC1806 cells treated with 2 nM PXT compared with the untreated control (Fig. S2C,E). No significant changes in cell migration were observed in MDA-MB-231 and MDA-MB-436 cells. These results indicate that alteration of αB-crystallin expression might be associated with the increase of cell migration induced by 5-FU in HCC1806 cells.

### 5-FU induces cell motility in cell lines with high αB-crystallin expression

**Figure 2:**
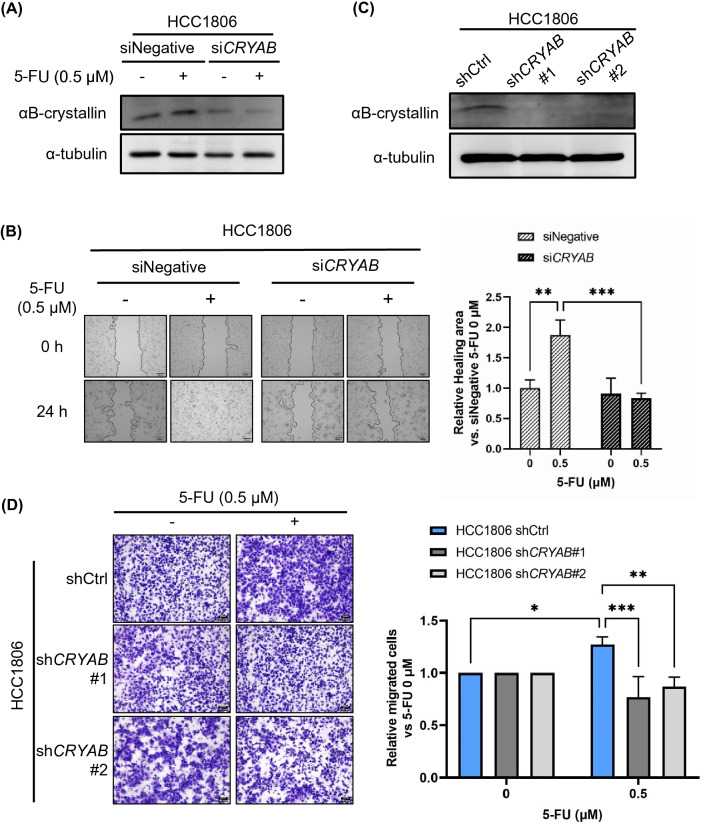
Silencing of αB-crystallin expression suppressed fluorouracil (5-FU)—induced increase of cell motility in HCC1806 cells. (**A**) HCC1806 cells were transfected with 10 nM of siRNA for 48 h and then treated with 0.5 μM 5-FU for 24 h. The protein expression level of αB-crystallin was evaluated by immunoblotting analysis. α-tubulin was used as the loading control. (**B**) HCC1806 cells were transfected with siRNA for 24 h and then treated with 0.5 μM 5-FU for 24 h. Images of the wound area at the beginning and end of the treatment period were recorded, and ImageJ software was used to calculate the relative healing area. siNegative: negative control siRNA; si*CRYAB*: siRNA targeting *CRYAB.* Scale bars = 100 μm. Data are presented as mean ± SD; n = 3. **P < 0.01; ***P < 0.001. (**C**) HCC1806 cells were infected with sh*CRYAB* by using a lentivirus and selected by puromycin. Stable cell lines harboring the negative control vector (shCtrl) or sh*CRYAB* (#1 or #2) were established. The protein expression level of αB-crystallin was evaluated by immunoblotting analysis; α-tubulin was used as the loading control. (**D**) Cells were treated or not with 0.5 μM 5-FU for 24 h and then added into Transwell inserts. The number of cells that had moved through the membrane was determined by using ImageJ software. shCtrl: negative control shRNA. Scale bars = 100 μm. Data are presented as mean ± SD; n = 3. *P < 0.05; **P < 0.01; ***P < 0.001. Results are representative of two independent experiments.

To examine our hypotheses that 5-FU increases cell motility in association with the expression of αB-crystallin, the cell migration experiments were repeated using HCC1806 cells in which the expression of αB-crystallin had been knocked down. Western blotting confirmed that 5-FU induced αB-crystallin expression and it was inhibited in si*CRYAB*-treated cells (Fig. 2A). In the wound healing assay, 0.5 μM 5-FU significantly increased cell migration in control HCC1806 cells but not in si*CRYAB*-treated cells (Fig. 2B). Similarly, 1 μM CDDP significantly increased cell migration in control HCC1806 cells but not in si*CRYAB*-treated cells (Fig. S3A,B). We also constructed two stable HCC1806 cell lines with low αB-crystallin expression by using shRNA (lines sh*CRYAB*#1 and sh*CRYAB*#2; Fig. 2C). A transwell migration assay revealed that 0.5 μM 5-FU significantly increased cell migration in HCC1806 cells transfected with negative control shRNA, and that this increase in cell migration was significantly ameliorated in HCC1806 cell lines sh*CRYAB*#1 and sh*CRYAB*#2 (Fig. 2D).

**Figure 3:**
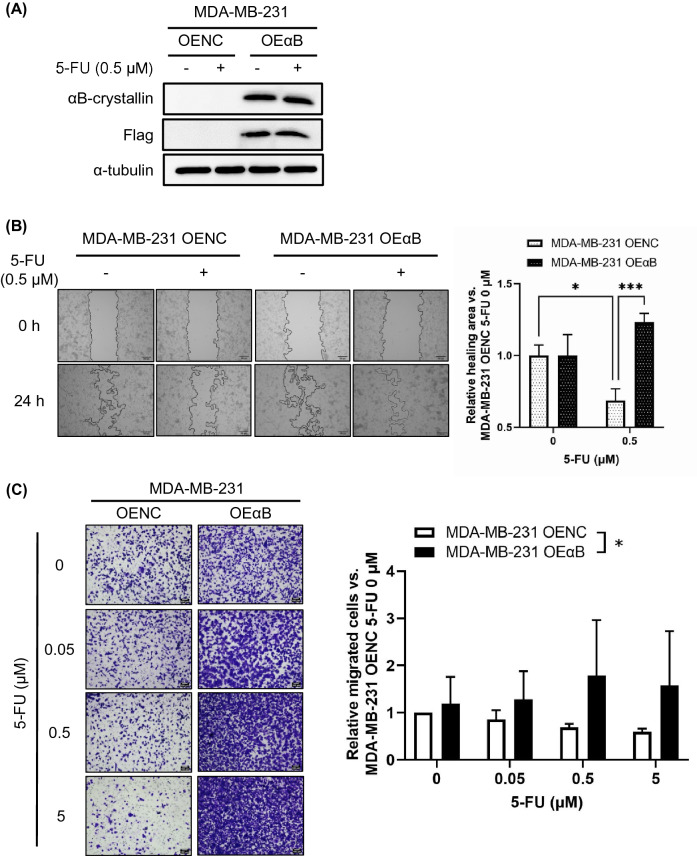
Fluorouracil (5-FU) increased cell motility in MDA-MB-231 cells overexpressing αB-crystallin. (**A**) MDA-MB-231 cells were transfected with αB-crystallin expression plasmid and selected by hygromycin. Stable cell lines harboring negative control vector (OENC) or showing αB-crystallin overexpression (OEαB) were treated with 0.5 μM 5-FU for 24 h. Protein expression was evaluated by immunoblotting analysis. Flag was used to tag αB-crystallin. α-Tubulin was used as the loading control. (**B**) Wound healing assay was performed by using MDA-MB-231 OENC cells and MDA-MB-231 OEαB cells treated with 0.5 μM 5-FU for 24 h. Images of the wound area at the beginning and end of the treatment period were recorded, and ImageJ was used to calculate the relative healing area. Scale bars = 100 μm. Data are presented as mean ± SD, n = 3. *P < 0.05; ***P < 0.001. (**C**) Cells were treated with 0, 0.05, 0.5, or 5 μM 5-FU for 24 h and then added into Transwell inserts. Following a further 24 h of incubation, non-migrated cells were removed and migrated cells in the bottom chamber were fixed. The number of cells that moved through the membrane was determined by using ImageJ. Scale bars = 100 μm. Data are presented as mean ± SD, n = 3. *P < 0.05. The results are representative of two independent experiments.

Moreover, we established a stable MDA-MB-231 cell line with high αB-crystallin expression (MDA-MB-231 OEαB cells) in which the expression of αB-crystallin was not affected by 5-FU (Fig. 3A). In the wound healing assay, 0.5 μM 5-FU significantly increased cell migration in MDA-MB-231 OEαB cells compared with that in MDA-MB-231 cells harboring the negative control vector (MDA-MB-231 OENC cells) (Fig. 3B). Besides, when treated with 0–5 μM 5-FU, the MDA-MB-231 OEαB cells showed significantly higher cell migration compared with MDA-MB-231 OENC cells (Fig. 3C). Similarly, in the wound healing assay, 0.5 μM CDDP significantly increased cell migration in MDA-MB-231 OEαB cells compared with that in MDA-MB-231 OENC cells (Fig. S3C,D). Together, these results indicate that 5-FU–induced cell migration of HCC1806 cells is mediated by αB-crystallin.

## Discussion

Systemic chemotherapy is often used for the treatment for TNBC and it is curative in most cases. However, a subset of patients develop distant metastasis after chemotherapy and face a higher risk of mortality due to changes in the tumor microenvironment of metastasis^21^, intratumor heterogeneity^22^, or chemotherapy-induced clonal evolution^23^. According to a database called METABRIC^24,25^, *CRYAB* expression level was tended to increase after chemotherapy treatment across breast cancer patients (Fig. S4A) and in the TNBC patients (Fig. S4B). Our present study proposed αB-crystallin is as one of the distinguishments of BL2 subtype of TNBC. Thus, considering the limitations of in vitro experiments, it is of interest for future work that αB-crystallin expression in BL2 type of TNBC patients before and after 5-FU treatment and that the correlation between αB-crystallin expression and therapy efficiency.

Here, we found that αB-crystallin is an intratumor factor that increases TNBC cell motility in response to treatment with 5-FU. It has been reported that αB-crystallin, which is activated by the nuclear factor-kappa B (NF-κB) signaling cascade, is required for cell migration and invasion in HER2-positive breast cancer cells^13^, and that 5-FU activates transcription factors such as NF-κB and hypoxia-inducible factor-1α in cancer cells^26,27^. Although further studies are needed to test the association of αB-crystallin with these transcription factors in TNBC cells, it is reasonable to postulate that αB-crystallin is upregulated by 5-FU through their activation.

Considering that PXT suppresses microtubule dynamic stability, which differs from 5-FU and CDDP that inhibit DNA synthesis, the increased cell motility after treatment with 5-FU and CDDP, but not with PXT in HCC1806 is associated with the cell microtubule cytoskeleton. Although the association between the cell microtubule cytoskeleton and αB-crystallin-mediated cell motility have not been reported in cancer cells, it has been reported in glial cells^28^, myoblasts^29^, cardiomyocytes^30^, and skeletal muscle^31^. One report has also clarified that αB-crystallin stimulates microtubule extension through a downstream RhoA/Rock signaling pathway^32^. Moreover, RhoA/Rock signaling pathway is crucial in the regulation of cancer cell motility by regulating cell cytoskeletal^33,34^. In a supplementary experiment, immunoblotting analysis verified the expression of RhoA in si*CRYAB*-treated HCC1806 cells was downregulated compared with that in control HCC1806 cells (data not shown). Meanwhile, the expression level of RhoA was not upregulated after 5-FU treatment according to the immunoblotting analysis. Hence, it is necessary to investigate the precise mechanism of RhoA in 5-FU-induced cell motility by evaluating the activity and intracellular localization of RhoA after 5-FU treatment in the future. Further exploration of the relationship between αB-crystallin and RhoA could lead to clarify the molecular mechanism of αB-crystallin–mediated cell motility.

In another supplementary experiment, we found that neither the silencing of αB-crystallin expression in HCC1806 cells nor the induction of overexpression of αB-crystallin in MDA-MB-231 cells had any effect on cell viability after treatment with 5-FU (data not shown). Although several studies have indicated that αB-crystallin is associated with cell viability through inhibition of apoptosis^35^ or anoikis^36^, our previous study showed no such effect on cell viability in SKBR3 cells treated with trastuzumab^37^, in which αB-crystallin was also detected. Combining this study with another of our previous studies that showed that αB-crystallin promoted microtubule formation of endothelial cells^37^, we surmise that αB-crystallin has specific functions in each subtype of breast cancer cell. Therefore, further studies are needed to fully clarify the role high expression of αB-crystallin plays in HCC1143 cells, which belong to the BL1 subtype of TNBC cells, and how the function of αB-crystallin is different in the BL2 subtype.

## Supplementary Information

Supplementary Information.

## Data Availability

All data generated or analyzed during this study are included in this published article.

## Abbreviations

BL2: Basal-like 2
HER2: Human epidermal growth factor receptor 2
MSL: Mesenchymal stem-like
NF-κB: Nuclear factor-kappa B
TNBC: Triple-negative breast cancer
