# Supplementary material for: Fluorouracil exacerbates alpha-crystallin B chain—mediated cell migration in triple-negative breast cancer cell lines
Source: Sci Rep. 2023 Mar 10;13:4010. doi: 10.1038/s41598-023-31186-7 (PMC10006185; doi:10.1038/s41598-023-31186-7)
Supplement: Supplementary file 1 — Supplementary Information. [file 41598_2023_31186_MOESM1_ESM.pptx]

## Slide 1
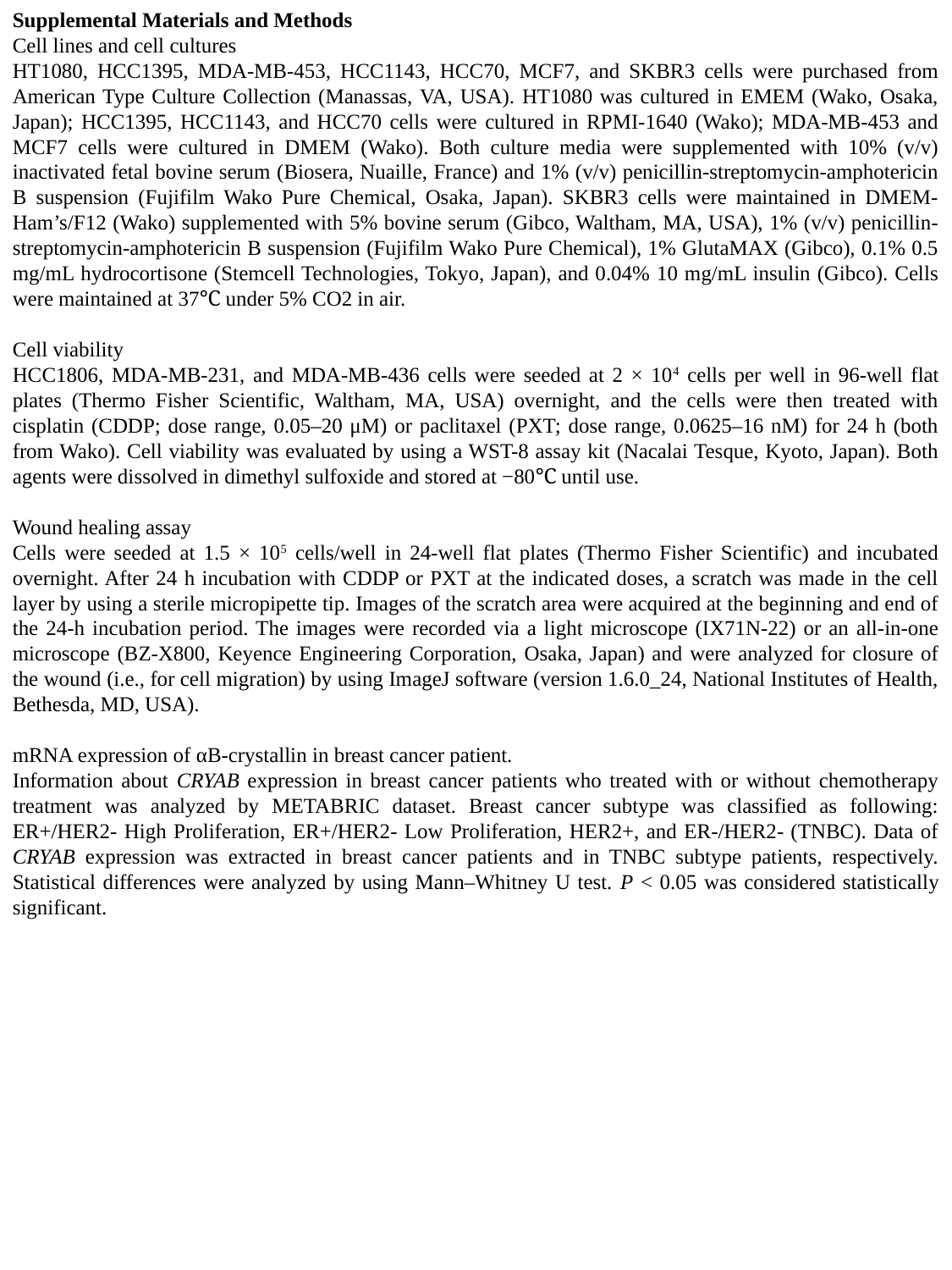

Supplemental Materials and Methods
Cell lines and cell cultures
HT1080, HCC1395, MDA-MB-453, HCC1143, HCC70, MCF7, and SKBR3 cells were purchased from American Type Culture Collection (Manassas, VA, USA). HT1080 was cultured in EMEM (Wako, Osaka, Japan); HCC1395, HCC1143, and HCC70 cells were cultured in RPMI-1640 (Wako); MDA-MB-453 and MCF7 cells were cultured in DMEM (Wako). Both culture media were supplemented with 10% (v/v) inactivated fetal bovine serum (Biosera, Nuaille, France) and 1% (v/v) penicillin-streptomycin-amphotericin B suspension (Fujifilm Wako Pure Chemical, Osaka, Japan). SKBR3 cells were maintained in DMEM-Ham’s/F12 (Wako) supplemented with 5% bovine serum (Gibco, Waltham, MA, USA), 1% (v/v) penicillin-streptomycin-amphotericin B suspension (Fujifilm Wako Pure Chemical), 1% GlutaMAX (Gibco), 0.1% 0.5 mg/mL hydrocortisone (Stemcell Technologies, Tokyo, Japan), and 0.04% 10 mg/mL insulin (Gibco). Cells were maintained at 37℃ under 5% CO2 in air.
Cell viability
HCC1806, MDA-MB-231, and MDA-MB-436 cells were seeded at 2 × 10^4^ cells per well in 96-well flat plates (Thermo Fisher Scientific, Waltham, MA, USA) overnight, and the cells were then treated with cisplatin (CDDP; dose range, 0.05–20 μM) or paclitaxel (PXT; dose range, 0.0625–16 nM) for 24 h (both from Wako). Cell viability was evaluated by using a WST-8 assay kit (Nacalai Tesque, Kyoto, Japan). Both agents were dissolved in dimethyl sulfoxide and stored at −80℃ until use.
Wound healing assay
Cells were seeded at 1.5 × 10^5^ cells/well in 24-well flat plates (Thermo Fisher Scientific) and incubated overnight. After 24 h incubation with CDDP or PXT at the indicated doses, a scratch was made in the cell layer by using a sterile micropipette tip. Images of the scratch area were acquired at the beginning and end of the 24-h incubation period. The images were recorded via a light microscope (IX71N-22) or an all-in-one microscope (BZ-X800, Keyence Engineering Corporation, Osaka, Japan) and were analyzed for closure of the wound (i.e., for cell migration) by using ImageJ software (version 1.6.0_24, National Institutes of Health, Bethesda, MD, USA).
mRNA expression of αB-crystallin in breast cancer patient.
Information about CRYAB expression in breast cancer patients who treated with or without chemotherapy treatment was analyzed by METABRIC dataset. Breast cancer subtype was classified as following: ER+/HER2- High Proliferation, ER+/HER2- Low Proliferation, HER2+, and ER-/HER2- (TNBC). Data of CRYAB expression was extracted in breast cancer patients and in TNBC subtype patients, respectively. Statistical differences were analyzed by using Mann–Whitney U test. P < 0.05 was considered statistically significant.

## Slide 2
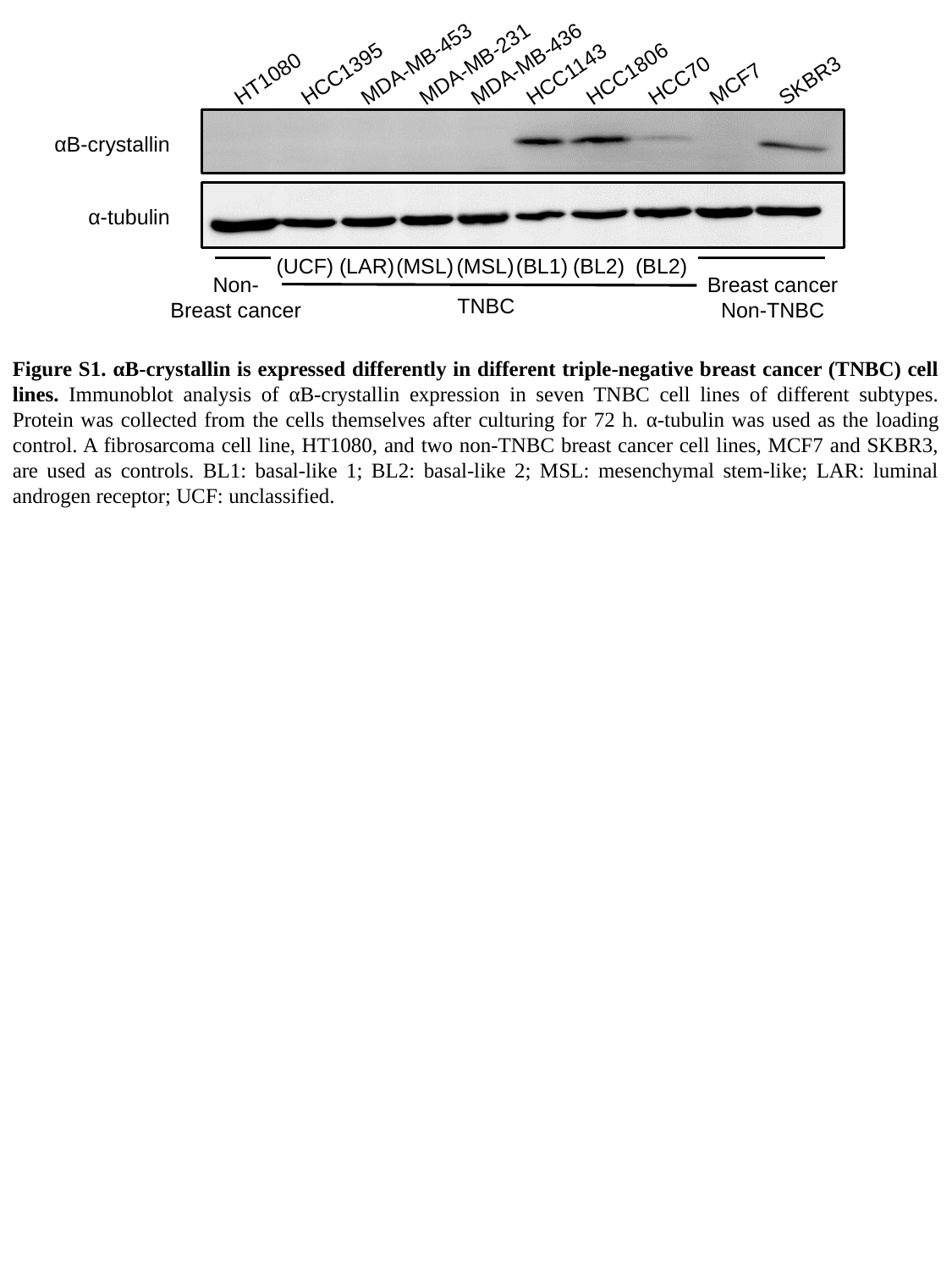

HCC1143
MDA-MB-231
HCC1806
MDA-MB-453
MDA-MB-436
HCC1395
HCC70
SKBR3
MCF7
HT1080
αB-crystallin
α-tubulin
(UCF)
(LAR)
(MSL)
(MSL)
(BL1)
(BL2)
(BL2)
Breast cancer
Non-TNBC
Non-
Breast cancer
TNBC
Figure S1. αB-crystallin is expressed differently in different triple-negative breast cancer (TNBC) cell lines. Immunoblot analysis of αB-crystallin expression in seven TNBC cell lines of different subtypes. Protein was collected from the cells themselves after culturing for 72 h. α-tubulin was used as the loading control. A fibrosarcoma cell line, HT1080, and two non-TNBC breast cancer cell lines, MCF7 and SKBR3, are used as controls. BL1: basal-like 1; BL2: basal-like 2; MSL: mesenchymal stem-like; LAR: luminal androgen receptor; UCF: unclassified.

## Slide 3
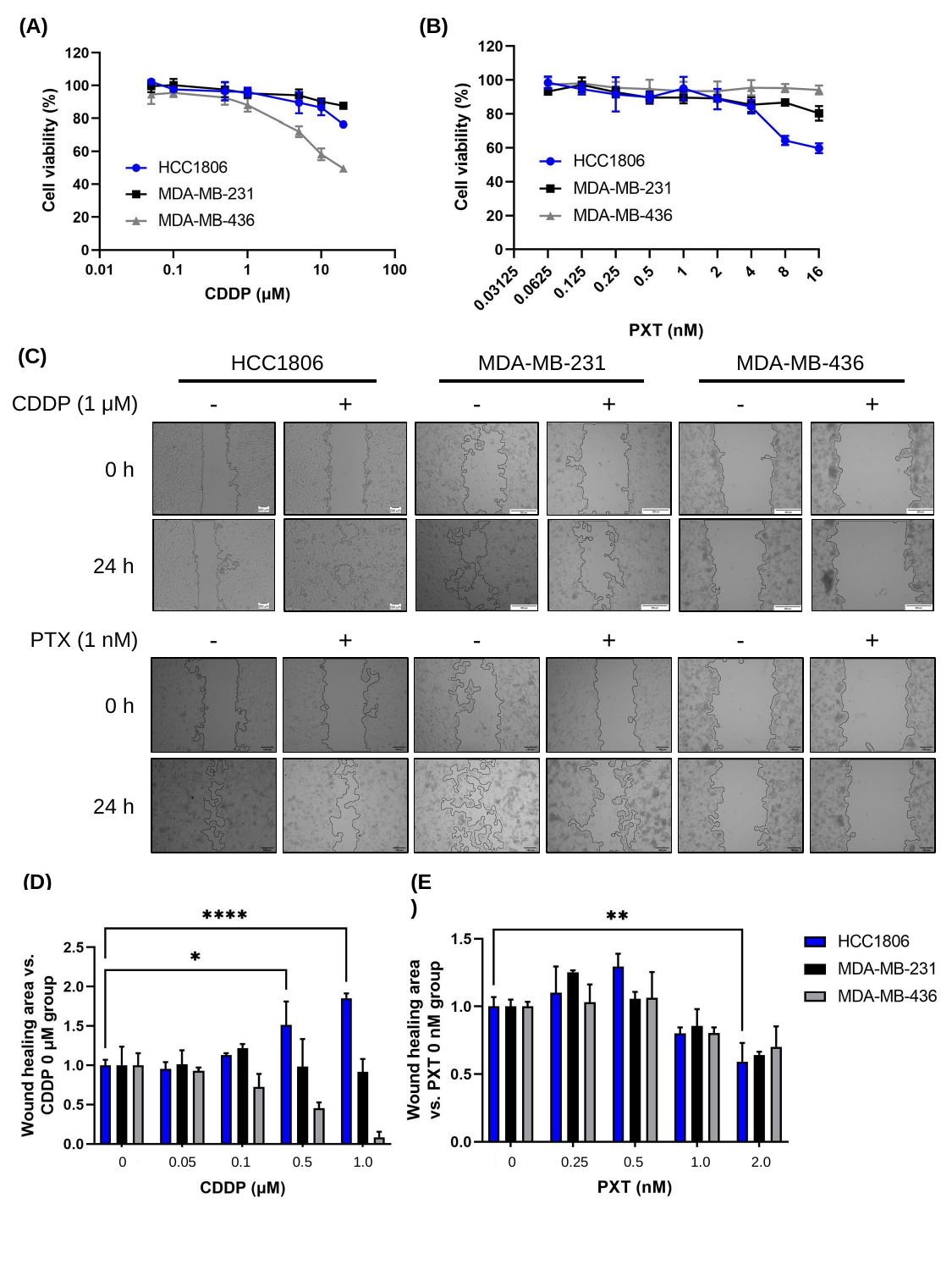

(A)
(B)
(C)
HCC1806
MDA-MB-231
MDA-MB-436
+
+
-
+
-
-
CDDP (1 μM)
0 h
24 h
+
+
-
+
-
-
PTX (1 nM)
0 h
24 h
(D)
(E)
0
0.05
0.1
0.5
1.0
0
0.25
0.5
1.0
2.0

## Slide 4
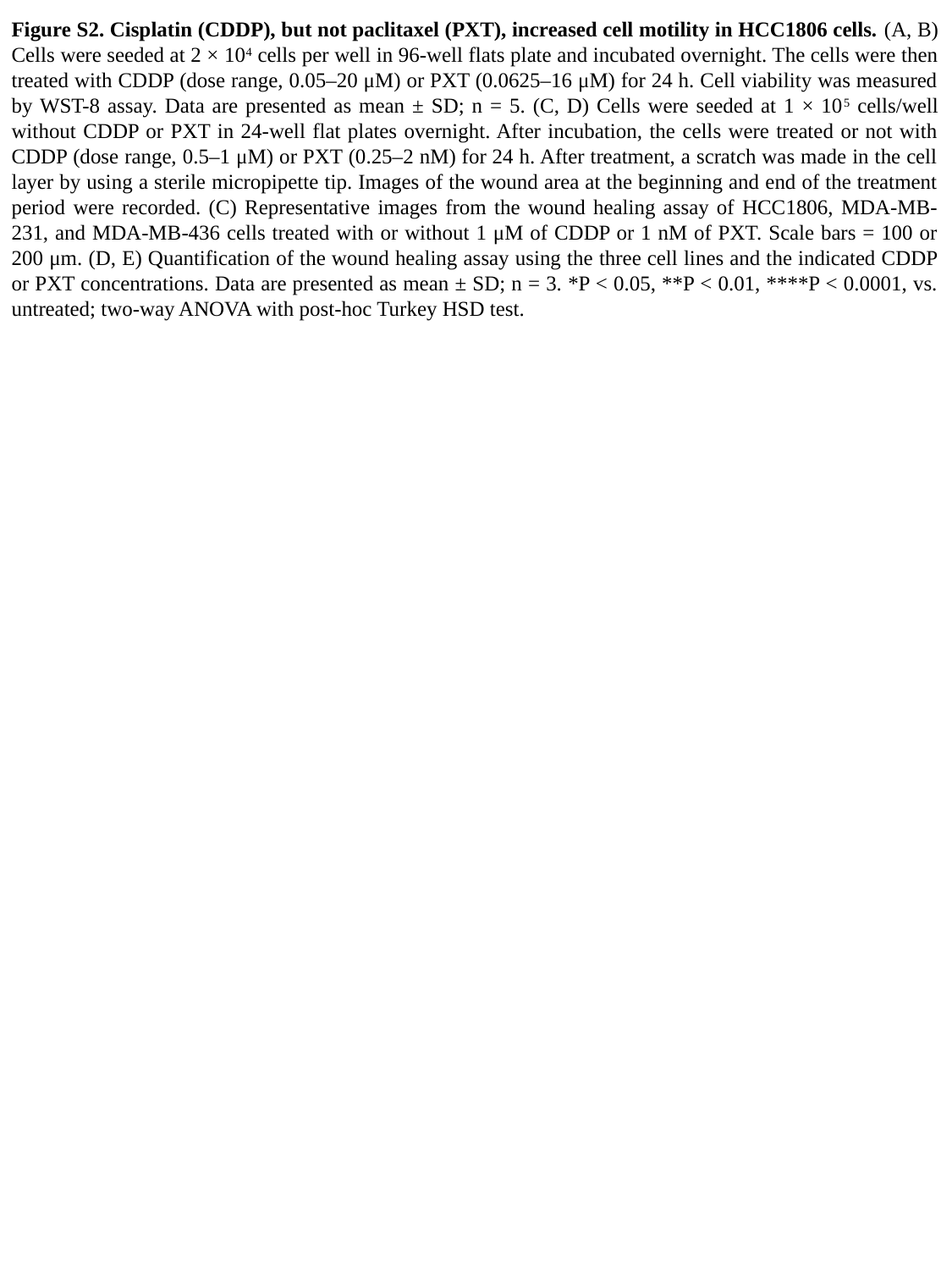

Figure S2. Cisplatin (CDDP), but not paclitaxel (PXT), increased cell motility in HCC1806 cells. (A, B) Cells were seeded at 2 × 10^4^ cells per well in 96-well flats plate and incubated overnight. The cells were then treated with CDDP (dose range, 0.05–20 μM) or PXT (0.0625–16 μM) for 24 h. Cell viability was measured by WST-8 assay. Data are presented as mean ± SD; n = 5. (C, D) Cells were seeded at 1 × 10^5^ cells/well without CDDP or PXT in 24-well flat plates overnight. After incubation, the cells were treated or not with CDDP (dose range, 0.5–1 μM) or PXT (0.25–2 nM) for 24 h. After treatment, a scratch was made in the cell layer by using a sterile micropipette tip. Images of the wound area at the beginning and end of the treatment period were recorded. (C) Representative images from the wound healing assay of HCC1806, MDA-MB-231, and MDA-MB-436 cells treated with or without 1 μM of CDDP or 1 nM of PXT. Scale bars = 100 or 200 μm. (D, E) Quantification of the wound healing assay using the three cell lines and the indicated CDDP or PXT concentrations. Data are presented as mean ± SD; n = 3. *P < 0.05, **P < 0.01, ****P < 0.0001, vs. untreated; two-way ANOVA with post-hoc Turkey HSD test.

## Slide 5
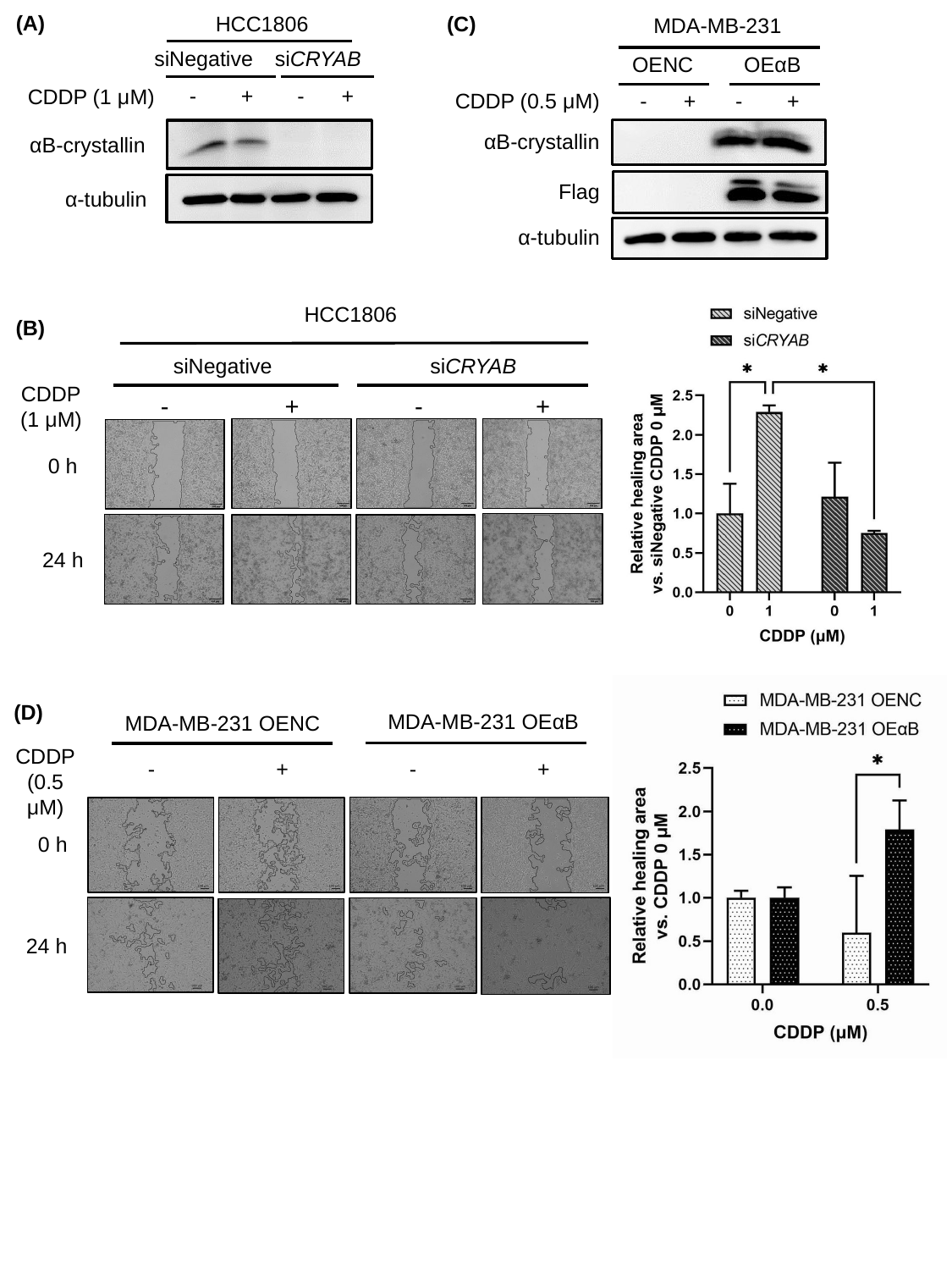

(A)
(C)
HCC1806
MDA-MB-231
siNegative
siCRYAB
OENC
OEαB
-
+
-
+
CDDP (1 μM)
CDDP (0.5 μM)
-
+
-
+
αB-crystallin
αB-crystallin
Flag
α-tubulin
α-tubulin
HCC1806
siNegative
siCRYAB
CDDP
(1 μM)
+
-
+
-
0 h
24 h
(B)
(D)
MDA-MB-231 OEαB
MDA-MB-231 OENC
CDDP
(0.5 μM)
-
+
-
+
0 h
24 h

## Slide 6
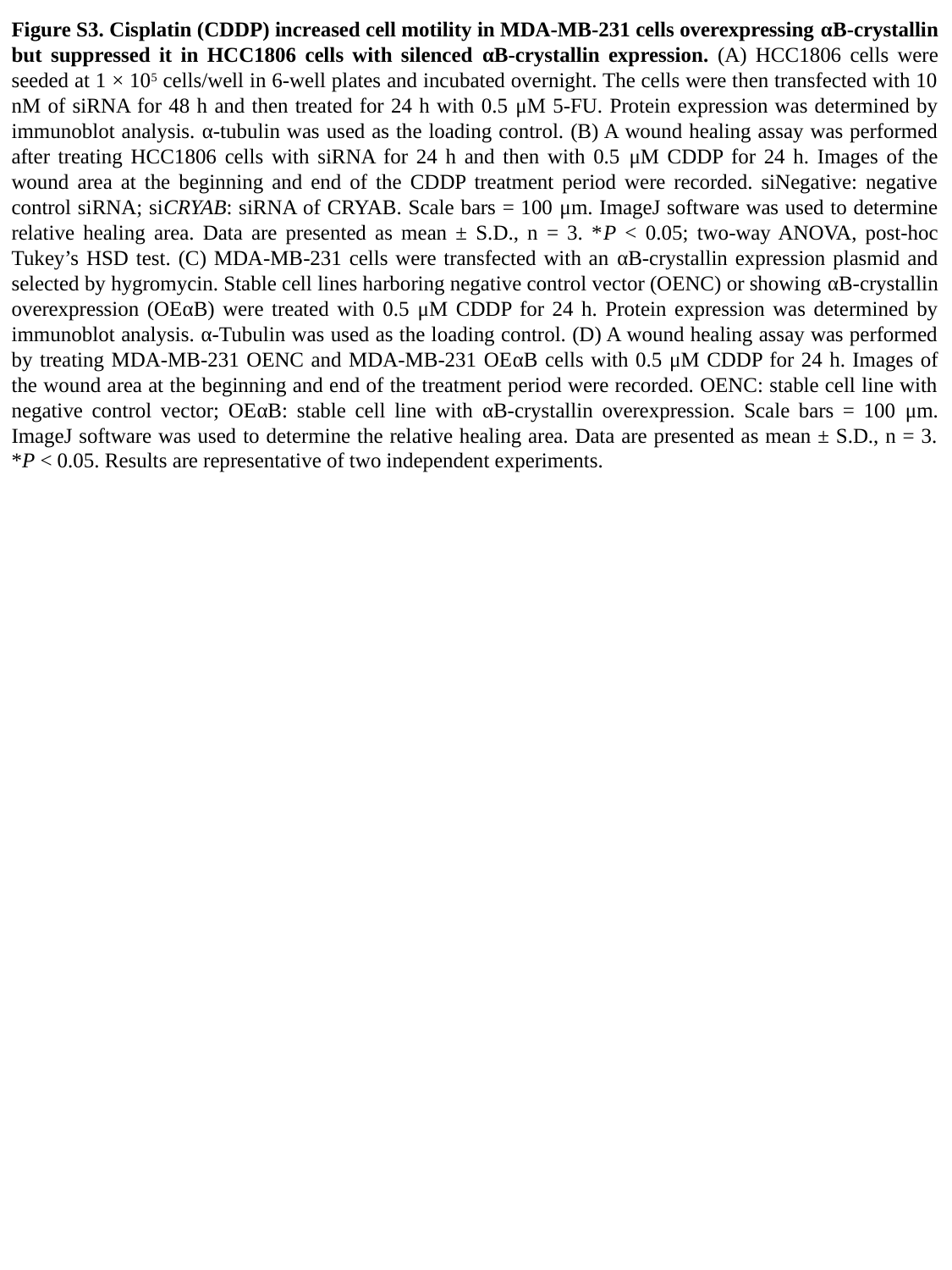

Figure S3. Cisplatin (CDDP) increased cell motility in MDA-MB-231 cells overexpressing αB-crystallin but suppressed it in HCC1806 cells with silenced αB-crystallin expression. (A) HCC1806 cells were seeded at 1 × 10^5^ cells/well in 6-well plates and incubated overnight. The cells were then transfected with 10 nM of siRNA for 48 h and then treated for 24 h with 0.5 μM 5-FU. Protein expression was determined by immunoblot analysis. α-tubulin was used as the loading control. (B) A wound healing assay was performed after treating HCC1806 cells with siRNA for 24 h and then with 0.5 μM CDDP for 24 h. Images of the wound area at the beginning and end of the CDDP treatment period were recorded. siNegative: negative control siRNA; siCRYAB: siRNA of CRYAB. Scale bars = 100 μm. ImageJ software was used to determine relative healing area. Data are presented as mean ± S.D., n = 3. *P < 0.05; two-way ANOVA, post-hoc Tukey’s HSD test. (C) MDA-MB-231 cells were transfected with an αB-crystallin expression plasmid and selected by hygromycin. Stable cell lines harboring negative control vector (OENC) or showing αB-crystallin overexpression (OEαB) were treated with 0.5 μM CDDP for 24 h. Protein expression was determined by immunoblot analysis. α-Tubulin was used as the loading control. (D) A wound healing assay was performed by treating MDA-MB-231 OENC and MDA-MB-231 OEαB cells with 0.5 μM CDDP for 24 h. Images of the wound area at the beginning and end of the treatment period were recorded. OENC: stable cell line with negative control vector; OEαB: stable cell line with αB-crystallin overexpression. Scale bars = 100 μm. ImageJ software was used to determine the relative healing area. Data are presented as mean ± S.D., n = 3. *P < 0.05. Results are representative of two independent experiments.

## Slide 7
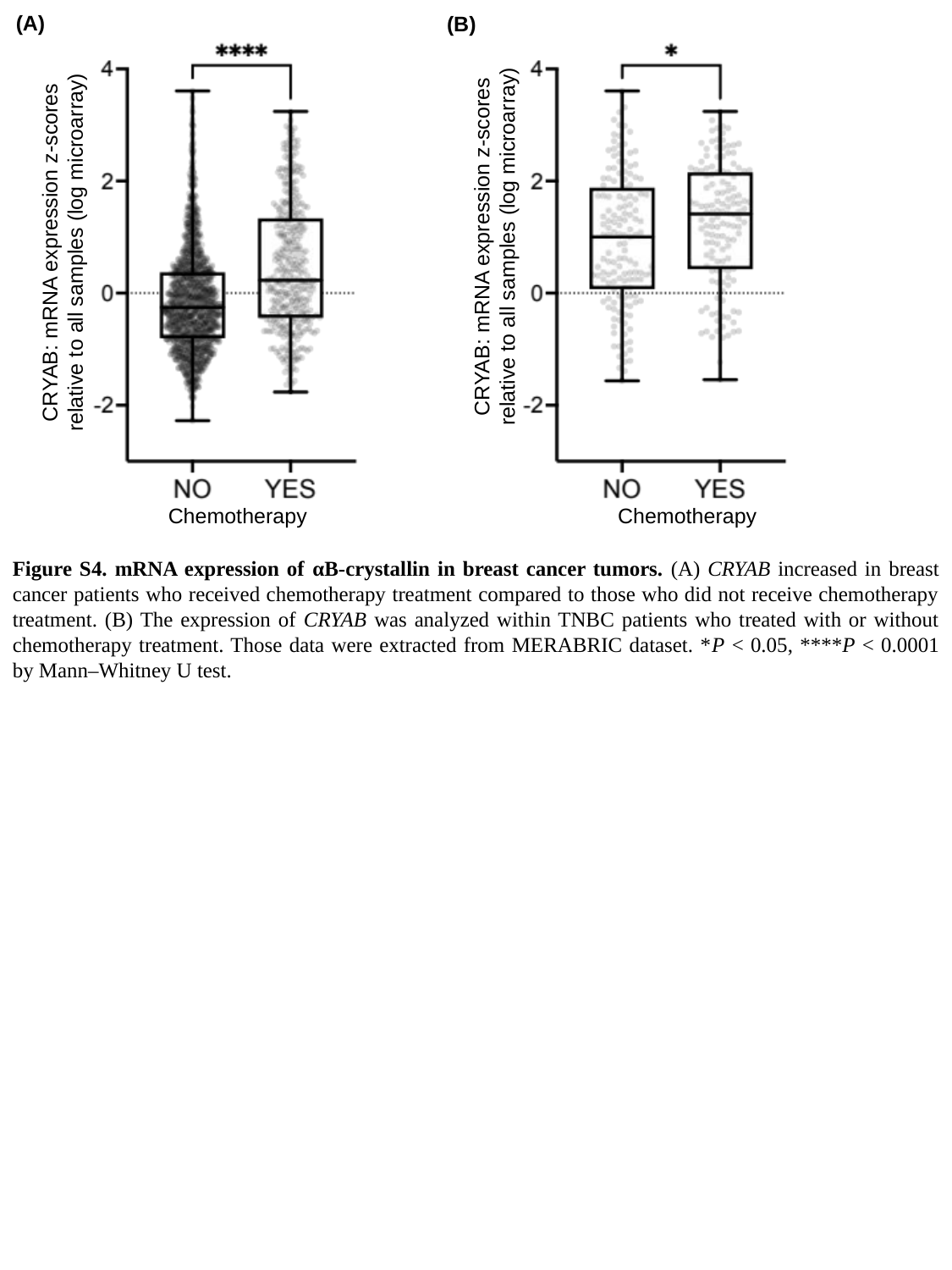

(A)
(B)
CRYAB: mRNA expression z-scores relative to all samples (log microarray)
CRYAB: mRNA expression z-scores relative to all samples (log microarray)
Chemotherapy
Chemotherapy
Figure S4. mRNA expression of αB-crystallin in breast cancer tumors. (A) CRYAB increased in breast cancer patients who received chemotherapy treatment compared to those who did not receive chemotherapy treatment. (B) The expression of CRYAB was analyzed within TNBC patients who treated with or without chemotherapy treatment. Those data were extracted from MERABRIC dataset. *P < 0.05, ****P < 0.0001 by Mann–Whitney U test.

## Slide 8
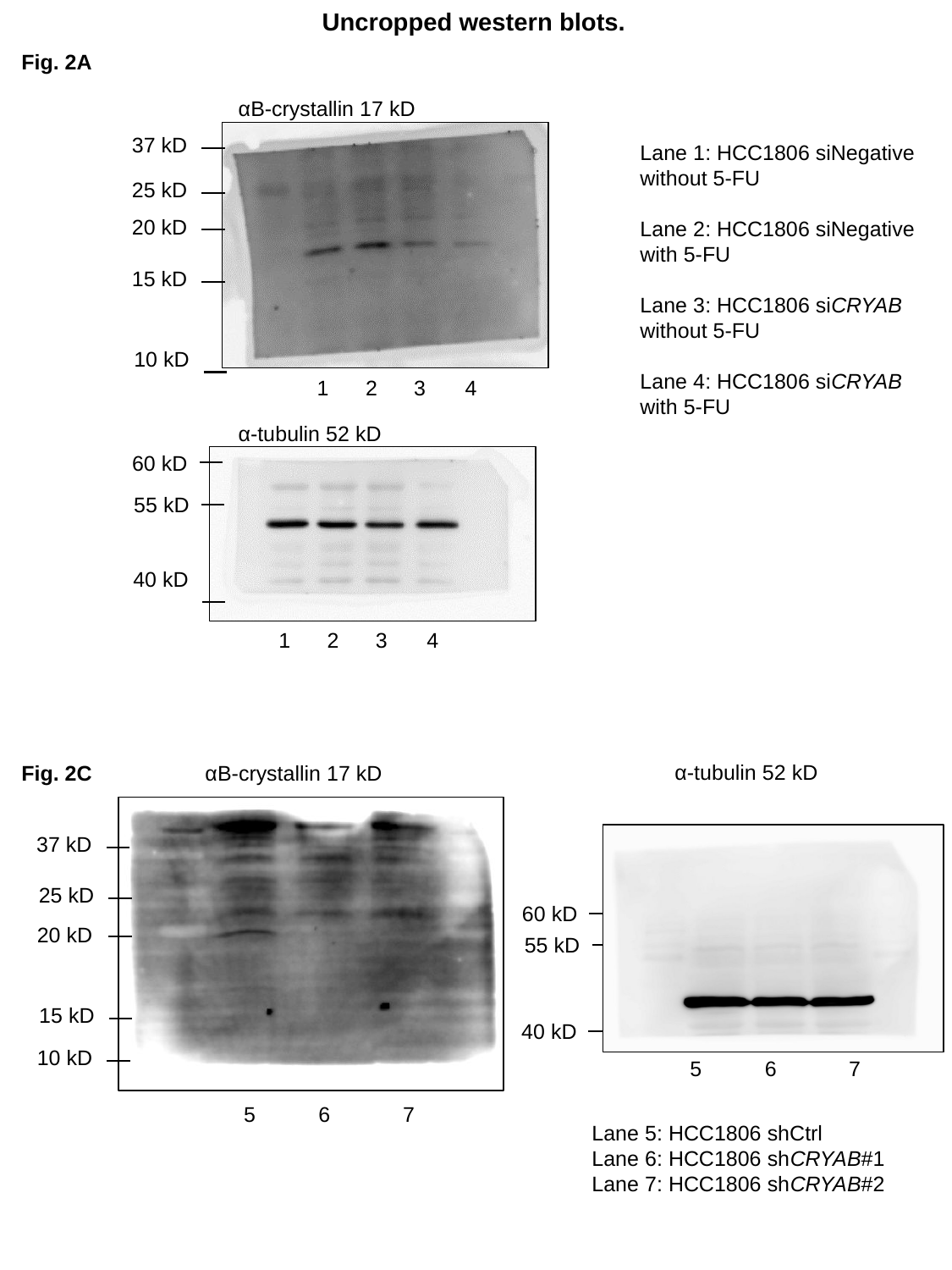

Uncropped western blots.
Fig. 2A
αB-crystallin 17 kD
37 kD
Lane 1: HCC1806 siNegative without 5-FU
Lane 2: HCC1806 siNegative with 5-FU
Lane 3: HCC1806 siCRYAB without 5-FU
Lane 4: HCC1806 siCRYAB with 5-FU
25 kD
20 kD
15 kD
10 kD
1
2
3
4
α-tubulin 52 kD
60 kD
55 kD
40 kD
1
2
3
4
α-tubulin 52 kD
αB-crystallin 17 kD
Fig. 2C
37 kD
25 kD
60 kD
20 kD
55 kD
15 kD
40 kD
10 kD
5
6
7
5
6
7
Lane 5: HCC1806 shCtrl
Lane 6: HCC1806 shCRYAB#1
Lane 7: HCC1806 shCRYAB#2

## Slide 9
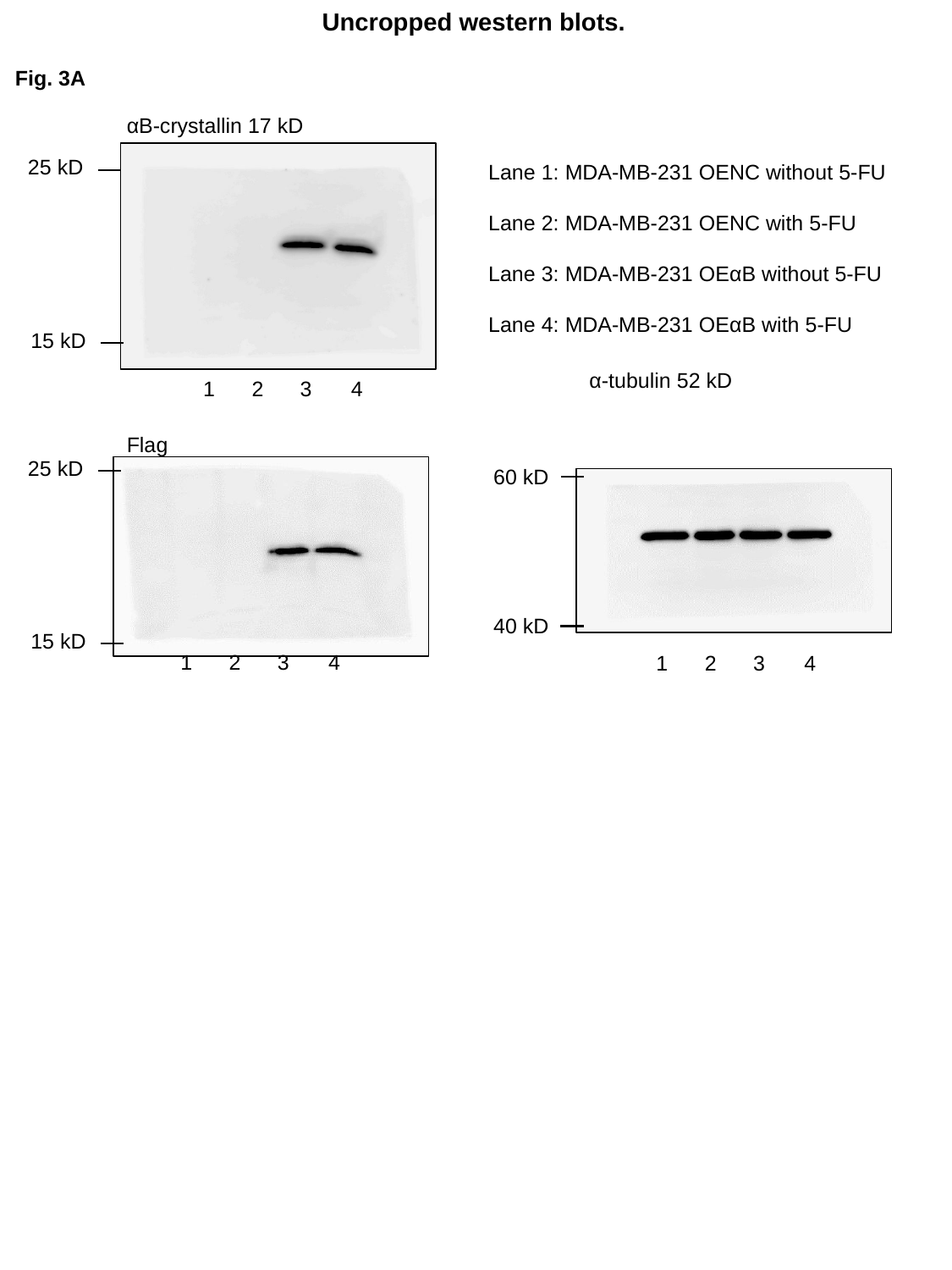

Uncropped western blots.
Fig. 3A
αB-crystallin 17 kD
25 kD
Lane 1: MDA-MB-231 OENC without 5-FU
Lane 2: MDA-MB-231 OENC with 5-FU
Lane 3: MDA-MB-231 OEαB without 5-FU
Lane 4: MDA-MB-231 OEαB with 5-FU
15 kD
α-tubulin 52 kD
1
2
3
4
Flag
25 kD
60 kD
40 kD
15 kD
1
2
3
4
1
2
3
4

## Slide 10
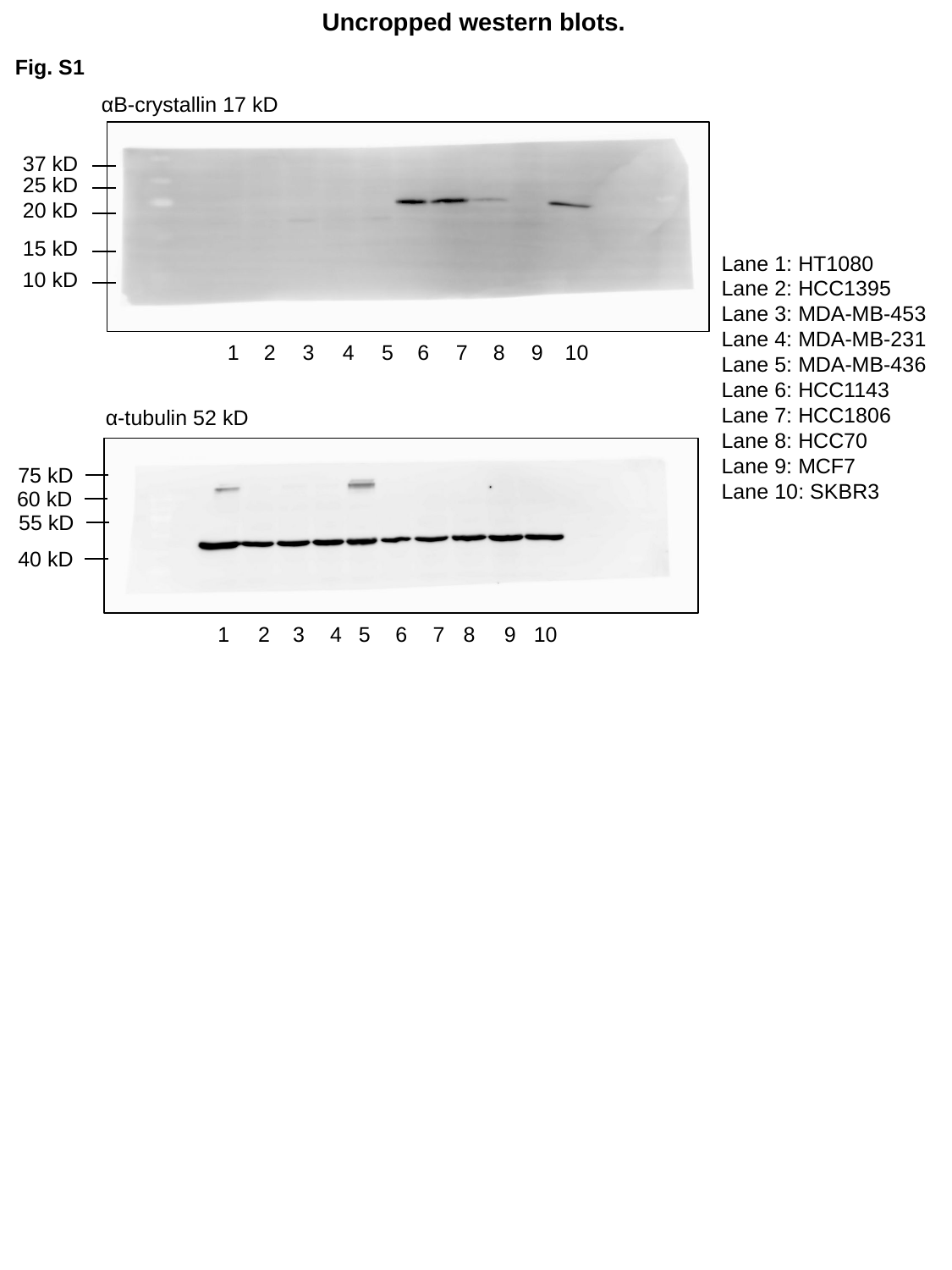

Uncropped western blots.
Fig. S1
αB-crystallin 17 kD
37 kD
25 kD
20 kD
15 kD
Lane 1: HT1080
Lane 2: HCC1395
Lane 3: MDA-MB-453
Lane 4: MDA-MB-231
Lane 5: MDA-MB-436
Lane 6: HCC1143
Lane 7: HCC1806
Lane 8: HCC70
Lane 9: MCF7
Lane 10: SKBR3
10 kD
1
2
3
4
5
6
7
8
9
10
α-tubulin 52 kD
75 kD
60 kD
55 kD
40 kD
1
2
3
4
5
6
7
8
9
10

## Slide 11
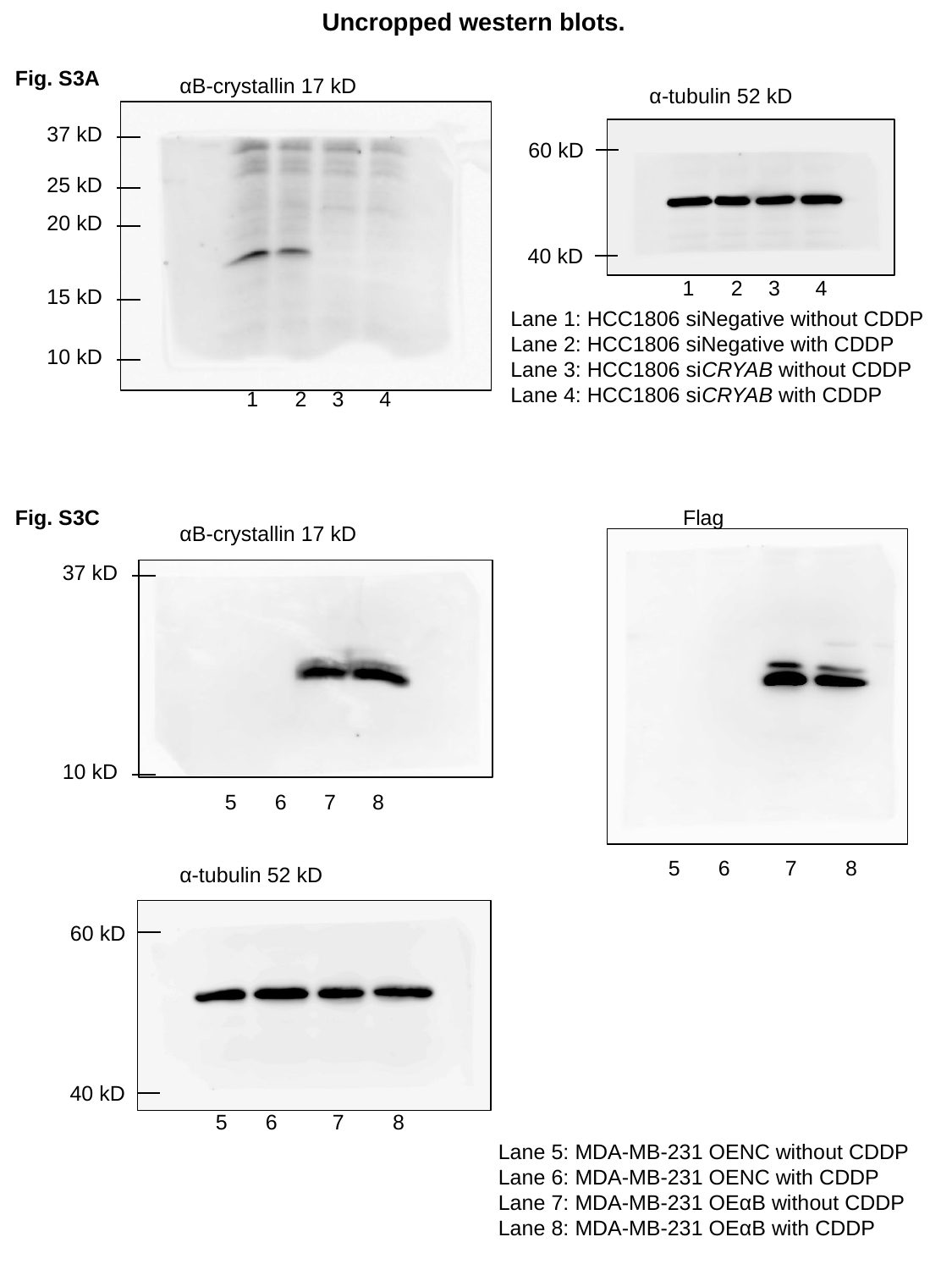

Uncropped western blots.
Fig. S3A
αB-crystallin 17 kD
α-tubulin 52 kD
37 kD
60 kD
25 kD
20 kD
40 kD
1
2
3
4
15 kD
Lane 1: HCC1806 siNegative without CDDP
Lane 2: HCC1806 siNegative with CDDP
Lane 3: HCC1806 siCRYAB without CDDP
Lane 4: HCC1806 siCRYAB with CDDP
10 kD
1
2
3
4
Flag
Fig. S3C
αB-crystallin 17 kD
37 kD
10 kD
5
6
7
8
5
6
7
8
α-tubulin 52 kD
60 kD
40 kD
5
6
7
8
Lane 5: MDA-MB-231 OENC without CDDP
Lane 6: MDA-MB-231 OENC with CDDP
Lane 7: MDA-MB-231 OEαB without CDDP
Lane 8: MDA-MB-231 OEαB with CDDP
